# Construction of optical spatiotemporal skyrmions

**DOI:** 10.1038/s41377-025-02028-0

**Published:** 2025-09-16

**Authors:** Houan Teng, Xin Liu, Nianjia Zhang, Haihao Fan, Guoliang Chen, Qian Cao, Jinzhan Zhong, Xinrui Lei, Qiwen Zhan

**Affiliations:** 1 School of Optical-Electrical and Computer Engineering, University of Shanghai for Science and Technology, Shanghai, China; 2 Shandong Provincial Engineering and Technical Center of Light Manipulations and Shandong Provincial Key Laboratory of Optics and Photonic Device, School of Physics and Electronics, Shandong Normal University, Jinan, China; 3 Collaborative Innovation Center of Light Manipulations and Applications, Shandong Normal University, Jinan, China; 4 Zhejiang Key Laboratory of 3D Micro/Nano Fabrication and Characterization, Department of Electronic and Information Engineering, School of Engineering, Westlake University, Hangzhou, Zhejiang China; 5 Westlake Institute for Optoelectronics, Fuyang, Hangzhou, China; 6 Zhangjiang Laboratory, Shanghai, China; 7 International Institute for Sustainability with Knotted Chiral Meta Matter (WPI-SKCM2), Hiroshima University, Higashihiroshima, Hiroshima, Japan

**Keywords:** Ultrafast photonics, Optical physics

## Abstract

The creation and manipulation of photonic skyrmions provide a novel degree of freedom for light-matter interactions, optical communication and nanometrology. Since the localized vortex within skyrmions arises from the twist and curl of the phase structure, the orbital angular momentum of light is essential for their construction. While numerous skyrmionic textures have been proposed, they are formed within the spatial domain and induced by the longitudinal orbital angular momentum. Here we theoretically propose and experimentally observe spatiotemporal skyrmions within a picosecond pulse wavepacket, generated through vectorial sculpturing of spatiotemporal wavepackets. The skyrmionic textures emerge within the spatiotemporal distribution of a vector field encompass all possible polarization states. Constructed upon the transverse orbital angular momentum, spatiotemporal skyrmions, in contrast to spatial skyrmions, exhibit no helical twisting perpendicular to the skyrmion plane, demonstrating potential stability against deformations or perturbations. These results expand the skyrmion family and offer new insights into optical quasiparticles, potentially leading to advanced applications in optical metrology, sensing, and data storage.

## Abstract (web summary)

We extend optical skyrmions from the spatial domain to the spatiotemporal domain, creating stable picosecond skyrmion wavepackets using transverse-OAM vectorial sculpturing.

## Graphical abstract

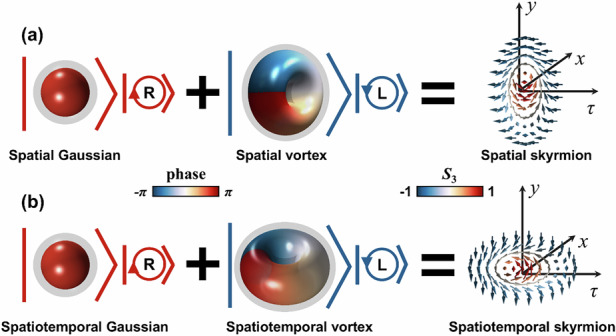

## Introduction

Skyrmions are topologically stable quasiparticles characterized by nontrivial textures, which cannot be continuously deformed into a trivial state due to their inherent, indestructible topological twist. Initially proposed within the theoretical framework of elementary particles^1–3^, skyrmions have subsequently emerged in diverse physical systems, including Bose-Einstein condensates^4–6^, nematic liquid crystals^7–9^, chiral magnets^10,11^, and water-wave^12,13^. The ultracompact size and inherent stability^14,15^, attributed to topological protection^16,17^, offer significant potential for applications in data storage and spintronic devices^18,19^. In recent years, skyrmionic textures have been introduced into the realm of optics^20–22^, where topological structures are realized through the precise manipulation of electromagnetic fields across various synthetic dimensions. On one hand, the spin-orbit interaction of light in nonparaxial beams offers a versatile platform for sculpting electromagnetic fields^22–24^. This dynamic mutual conversion between angular momentum components facilitates the formation of spin or field skyrmions^22,25–28^. On the other hand, Stokes skyrmions have been generated in paraxial beams^29–40^, where the topological features are encoded within the polarization texture of structured light^41,42^ and manipulated through Poincaré beams^43^, providing a novel pathway to emulate diversified topologies in higher-dimensional light fields^44,45^. The robustness of photonic skyrmions, demonstrated through their resilience to complex polarization aberrations^46^, suggests their potential for applications in nanoscale^47,48^, optical communications^49^, and photonic computing^50^.

**Fig. 1:**
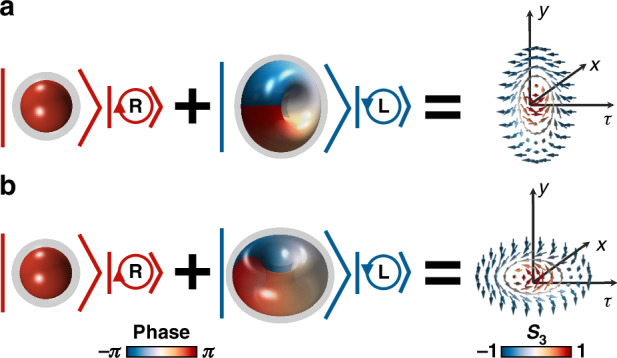
Spatial and spatiotemporal skyrmions. **a** Schematic of an optical spatial skyrmion constructed by a pair of spatial Laguerre–Gaussian (LG) modes with orthogonal polarization states. The normal vector of the spatial skyrmion plane is parallel to the propagation direction. **b** Schematic of an optical spatiotemporal skyrmion wavepacket, constructed by a pair of spatiotemporal Laguerre–Gaussian (LGst) modes with orthogonal polarization states. In contrast to the former, the normal vector of the spatiotemporal skyrmion plane is perpendicular to the propagation direction. The scalar spatiotemporal LGst modes could be constructed by spatiotemporal hologram^52,53^. The colormap of the intensity iso-surfaces corresponds to the phases of LG (or LGst) modes, respectively. The skyrmion texture can be visualized via a mapping (e.g., stereographic projection) from a parameter space (such as the Poincaré sphere) to real space (e.g., a spatial plane or a spatiotemporal plane)

The orbital angular momentum (OAM) of light plays a crucial role in constructing skyrmionic textures in both paraxial and nonparaxial beams^22,29,30^, as the localized vortex within these topological quasiparticles arises from the twist and curl of the phase structure. Of the various skyrmionic objects proposed in optical systems, they are formed within the spatial domain, which can be regarded as ‘longitudinal skyrmions’ induced by the longitudinal OAM, where the skyrmion plane is perpendicular to the propagation direction, and the localized spin structures remain static over time [Fig. 1a]. This results in a helical twist within the skyrmion tube, attributed to the Gouy phase shift among propagation. Transverse OAM embedded within pulsed wavepackets, on the other hand, provides an additional degree of freedom for manipulating light topology^51^. Driven by advancements in ultrafast lasers, spectral manipulation, and pulse characterization techniques, spatiotemporal sculpting of light has become feasible within time-dependent optical pulses^51–58^. While numerous topological structures have been observed in time-varying electromagnetic excitations, such as toroidal light pulses^59–62^ and optical hopfions^63^, these structures primarily reside within the spatial domain and their topological properties are limited to scalar fields. Vectorial topological quasiparticles in the spatiotemporal domain remain largely unexplored.

In this work, we theoretically propose and experimentally demonstrate optical spatiotemporal skyrmions in the space-time domain, achieved through the utilization of transverse OAM and vectorial shaping of pulsed light. By extending vectorial control from a purely spatial framework to a fully spatiotemporal paradigm, we construct the fundamental spatiotemporal skyrmion texture using spatiotemporal Gaussian mode pulses and spatiotemporal vortex mode pulses with orthogonal polarization states, which are carefully aligned both temporally and spatially [Fig. 1b]. The spatiotemporal skyrmion textures emerge within the vector field’s spatiotemporal distribution, encompassing all possible polarization states across both the time and spatial dimensions. In these textures, the skyrmions exhibit no helical twisting perpendicular to the skyrmion plane (*t-x* plane) due to the uniform vector sculpting along the *y*-axis, resulting in a perfect skyrmion tube with an identical skyrmion slice. The helicity of spatiotemporal skyrmion gradually evolves upon propagation caused by the interplay between medium dispersion and spatial diffraction. Since transient spatiotemporal skyrmions maintain a stable topological texture in the spatiotemporal dimension, they hold significant potential for nontrivial light-matter interactions and offer additional degrees of freedom for information transfer.

## Results

### Theoretical analysis and numerical simulations

The optical pulse wavepackets can be expressed as the product of the carrier wave and an envelope function as $E(t,x,y,z)=\varPsi (t,x,y,z)\exp (i{\omega }_{0}t-ikz)$, where ${\omega }_{0}={k}_{0}c=2\pi c/{\lambda }_{0}$ is the central angular frequency and *k*_0_ is the wavenumber in a vacuum. Within the framework of the scalar, paraxial, and narrow bandwidth approximations, the evolution of the envelope wavepacket in a uniform dispersive medium is governed by^57–59^

1 $$\frac{{\partial }^{2}\varPsi }{\partial {x}^{2}}+\frac{{\partial }^{2}\varPsi }{\partial {y}^{2}}-{k}_{0}{\beta }_{2}\frac{{\partial }^{2}\varPsi }{\partial {\tau }^{2}}+2{k}_{0}i\frac{\partial \varPsi }{\partial z}=0$$

where Ψ is the scalar envelope function, *τ* = *t* – *z*/*v*_g_ is the normalized local time, *z* is the propagation distance, *v*_g_ is the group velocity, *β*_2_ is the group velocity dispersion (GVD) coefficient of the dispersive medium. In an anomalous GVD medium with *β*_2_ = − 1/*k*_0_ (assuming the wavepacket is uniformly distributed along *y*-direction). Equation (1) admits a closed-form solution as a LGst wavepacket (at *z* = 0), which could be expressed as^53,59^

2 $${\varPsi }_{\mathrm{STLG}}(\tau ,x)\propto {\left(\frac{\sqrt{2({\gamma }^{2}{\tau }^{2}+{x}^{2})}}{{w}_{0}}\right)}^{|l|}\exp \left(-\frac{{\gamma }^{2}{\tau }^{2}+{x}^{2}}{{w}_{0}^{2}}\right){L}_{p}^{|l|}\left(\frac{2({\gamma }^{2}{\tau }^{2}+{x}^{2})}{{w}_{0}^{2}}\right)\exp \left[il{\tan }^{-1}\left(\frac{x}{\gamma \tau }\right)\right]$$

where ${w}_{0}$ is the waist radius; ${L}_{p}^{|l|}$ is the associated Laguerre polynomials of the order *p* and *l*. *γ* is a scaling factor of normalized local time.

Equation (2) describes the spatiotemporal LGst mode as the scalar solution of Eq. (1). Maxwell’s equations are inherently vectorial, describing the electric field as comprising three components: *E*_*x*_, *E*_*y*_, and *E*_*z*_. For a monochromatic light propagating along the *z*-direction, and under the paraxial approximation, only the transverse components *E*_*x*_ and *E*_*y*_ are typically considered. These components define the spatially varying polarization state of light, known as vector light, a phenomenon extensively studied both theoretically and experimentally. However, the polarization dynamics of pulsed light, varying with both time and space, remain largely unexplored. Here, we introduce a wavepacket with spatiotemporally evolving polarization, whose Stokes parameters form a distinctive topological structure—a skyrmion—in the *τ-x* plane. By scanning the time slice at a fixed longitudinal position, the instantaneous Stokes vectors are measured to construct the polarization distribution in the (*τ*, *x*) plane and reconstruct the spatiotemporal skyrmion.

In monochromatic light, the spatial skyrmion topology can be formed by a pair of spatial LG modes with orthogonal polarization states^29,30,36^ as ${\bf{E}}(x,y)\propto L{G}_{0,0}(x,y){{\bf{e}}}_{R}+L{G}_{0,1}(x,y){{\bf{e}}}_{L}$. In pulsed light, the spatiotemporal skyrmion topology pulse can be formed by a pair of LGst pulses with orthogonal polarization states, which have the following expression

3 $${\bf{E}}(\tau ,x,y)\propto \exp \left(-\frac{{y}^{2}}{{y}_{0}^{2}}\right)[\cos \,{c}_{0}\,LGs{t}_{0,0}(\tau ,x){{\bf{e}}}_{R}+\,\sin \,{c}_{0}\,{e}^{i{\phi }_{\gamma }}LGs{t}_{0,1}(\tau ,x){{\bf{e}}}_{L}]$$

where ${{\bf{e}}}_{R}=\frac{1}{\sqrt{2}}({{\bf{e}}}_{X}+i{{\bf{e}}}_{Y})$,${{\bf{e}}}_{L}=\frac{1}{\sqrt{2}}({{\bf{e}}}_{X}-i{{\bf{e}}}_{Y})$ represent right-circular polarization (RCP) and left-circular polarization (LCP) components of transverse electric field, and LGst_*p,l*_ is the LGst mode characterized by radial and azimuthal indices *p*, *l*. The global phase difference between the two orthogonal polarization components is denoted by *ϕ*_γ_, which controls the helicity textures of topological quasiparticle. *c*_0_ is a constant that determines the amplitude ratio of the orthogonal modes. *y*_0_ is the spatial width of the wavepacket along the *y* direction.

**Fig. 2:**
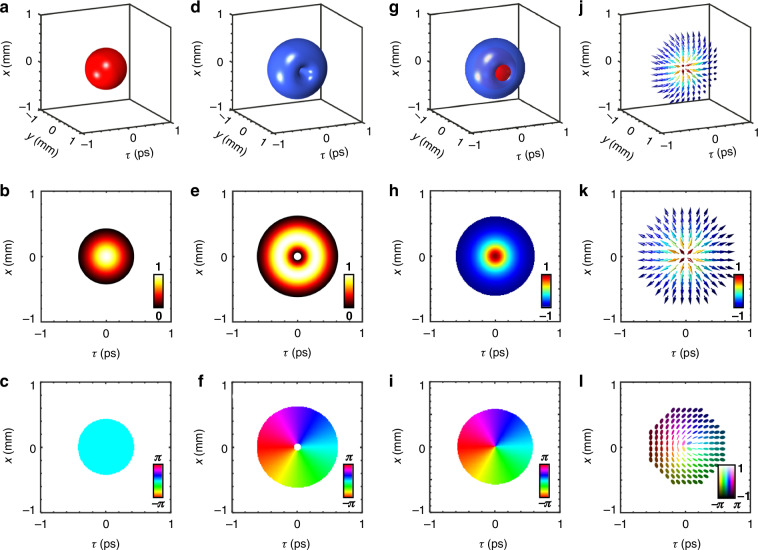
Simulation results of spatiotemporal skyrmion topology. **a** 3D iso-intensity (10% max) profiles of RCP spatiotemporal wavepacket with the corresponding **b** sliced intensity and **c** phase patterns at *y* = 0 plane. **d**–**f** 3D iso-intensity (10% max) profiles and corresponding sliced intensity and phase patterns (at *y* = 0 plane) of the LCP component. **g** A mixed 3D iso-intensity (10% max) profile of RCP and LCP spatiotemporal wavepacket. The normalized Stokes distribution of **h** out-of-plane (S_3_) and **i** in-plane component [tan^−1^(S_1_/S_2_)]. **j** The 3D optical Stokes orientation distribution. **k** The top view of Stokes orientation distribution. **l** The elliptical polarization distributions of spatiotemporal skyrmion topology. The Hue color denotes the polarization azimuth, and brightness represents ellipticity from left- to right-handed circular states. The definition and calculation of the Stokes parameters are detailed in the Methods section

There are two typical methods for generating vector beams. The first method involves controlling the spatial distribution of the polarization state by manipulating the amplitude ratio and phase difference between the LCP and RCP components^30^. The second method exploits the properties of waveplates, which can rotate the polarization ellipse within the Stokes space^36,64,65^. Consequently, spatially varying waveplates can modify the spatial distribution of the polarization state^36,56^. In our work, spatiotemporal polarization distribution is realized by employing the first method. A spatiotemporal vortex wavepacket of LGst_0,1_ mode [Fig. 2d] and a Gaussian wavepacket of LGst_0,0_ mode [Fig. 2a] are first produced, corresponding to LCP and RCP polarization, respectively. When the two wavepackets are carefully coaxially superimposed in the space-time plane [Fig. 2g], the spatiotemporal polarization distribution is determined by the amplitude ratio and phase difference between the RCP and LCP components. The amplitude and phase distributions of their cross-sections of RCP and LCP (*y* = 0 plane) are shown in Fig. 2b, c e, f. At the center of the wave packet (*y* = 0 plane), the amplitude ratio of the Gaussian mode (LGst_0,0_) to the vortex mode (LGst_0,1_) is maximal, resulting in right-handed polarization. At the edges (*y* = 0 plane), this ratio is minimal, producing left-handed polarization. The intensity ratio decreases continuously from infinity at the center of the wavepacket to zero at its edge along the radial direction of *y* = 0 plane. At a specific radius where the Gaussian and vortex mode amplitudes are equal, linear polarization is observed. The helicity of skyrmion is controlled by the phase difference between the RCP and LCP components. Since the Gaussian mode has a constant phase, this phase difference is entirely determined by the vortex phase, which defines the orientation angle of the linear polarization. The spatiotemporal polarization distribution can be depicted using a polarization ellipse [Fig. 2l] or Stokes parameters [Fig. 2j, k]. It should be note that the polarization is defined conventionally from the transverse field components *E*_*x*_(*τ*) and *E*_*y*_(*τ*), sampled over time at a fixed longitudinal position. The out-of-plane and in-plane components of the Stokes parameters are shown in Fig. 2h, i, respectively. The out-of-plane component gradually transitions from 1 to −1 along the radial direction [Fig. 2h]. Meanwhile, the in-plane component, represented by [tan^−1^(S_1_/S_2_)], undergoes a full 2*π* rotation along the azimuthal direction [Fig. 2i]. The Stokes orientation distribution in *τ*-*x* plane clearly illustrate the skyrmion topological texture [Fig. 2j, k]. Through stereographic projection, the normalized reduced Stokes parameters in the spatiotemporal plane are mapped onto the Poincaré sphere, covering it exactly once and yielding a skyrmion number of 1.

### Experimental results

Utilizing the Fourier transform relationship between the time-space domain (*τ*, *x*) and the spatiotemporal frequency domain (*Ω*, *ξ*), we construct a corresponding spatial spectrum to generate an LGst beam in the space-time domain^53,59^. Accordingly, we experimentally synthesize the spatiotemporal skyrmion wavepackets by 2D spatial light modulation and ultrafast pulse shaping^51,59,66^.

**Fig. 3:**
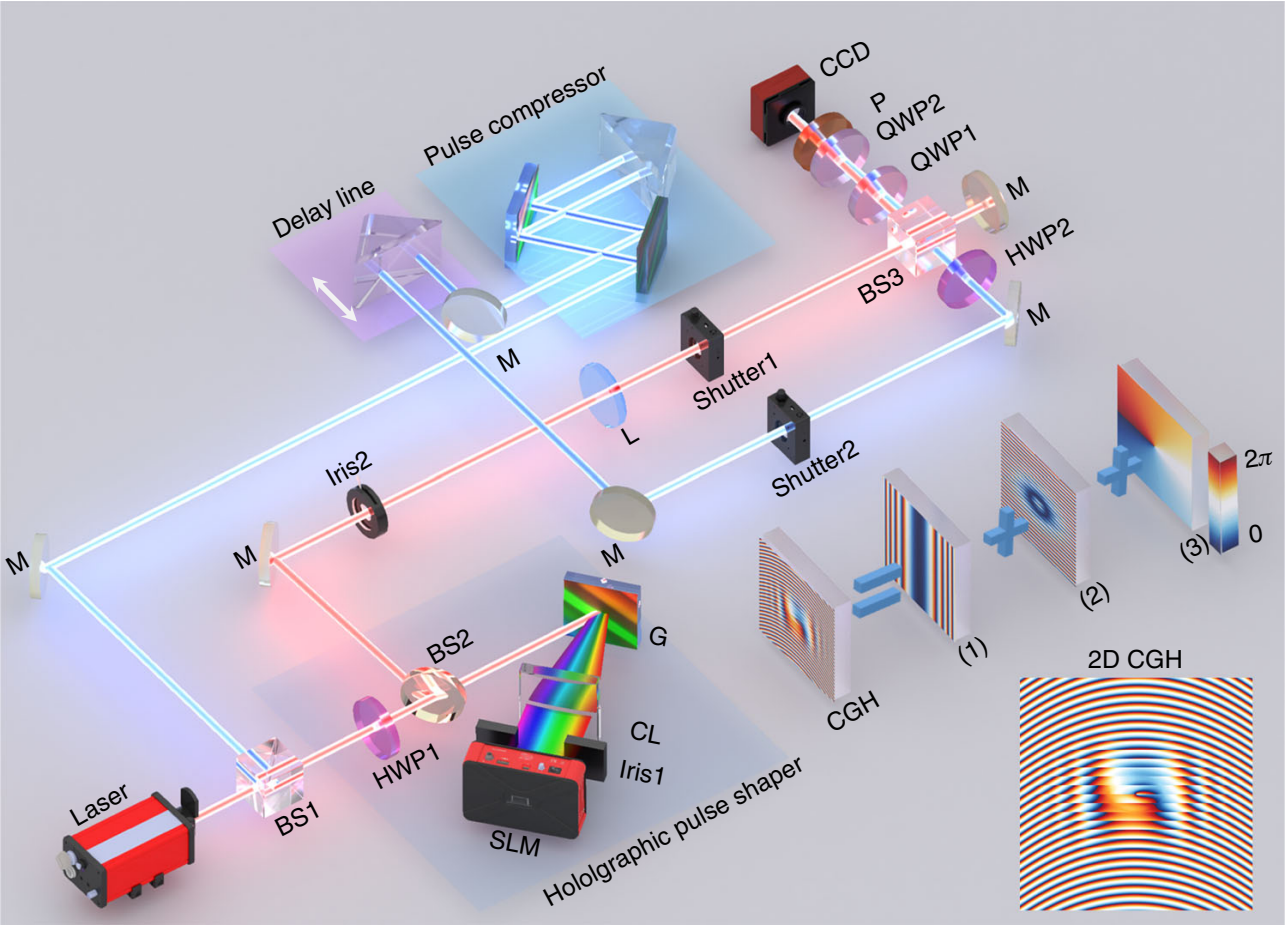
Optical setup for synthesizing and characterizing vectoral spatiotemporal skyrmion wavepackets. The apparatus comprises three components: (i) a 2D vectorial ultrafast holographic pulse shaper that incorporates a diffraction grating, a cylindrical lens, and an SLM, modulating only the *x*-polarization while leaving the y-polarization unaffected to control the amplitude ratio and phase difference between the *x*- and *y*-polarizations, thereby enabling polarization distribution control; (ii) a pulse compression system employing a parallel grating pair; and (iii) a time delay line system for fully reconstructing the 3D profile of the generated spatiotemporal wavepacket. Spatiotemporal holography used here must achieve accurate complex amplitude modulation in the zeroth order to ensure precise coaxial alignment between the *x*- and *y*-polarizations. The synthesized computer-generated hologram (CGH) enables complex-amplitude modulation of *x*-polarization, where the grating phase depth and delay regulate the amplitude and phase of the *x*-polarization component of incident beam, respectively^52,53,67^. The CGH embedded in the SLM consists of three components: (1) the phase distribution of a spatial-spectral LGst_01_ mode; (2) a GDD phase for managing the pre-chirp of the input pulse and balancing diffraction and dispersion; and (3) a phase-only diffraction grating with a phase depth modulated by the magnitude of Eq. (2) (see Methods). The 2D images of the composite CGH is placed in the bottom-right corner. BS beam splitter, HWP half-wave plate, QWP quarter-wave plate, P polarizer, L lens, M mirror, G grating, CL cylindrical lens, SLM spatial light modulator

Schematic illustration of the experimental setup is shown in Fig. 3. A chirped horizontally polarized pulsed beam is divided into an object beam and a probe beam by a polarization beamsplitter (BS1). The object beam was polarized at 45° by HWP1, and then spatially spread along *y*-axis by a grating and collimated onto a spatial light modulator (SLM) through a cylindrical lens. The SLM plane can be regarded as the Fourier conjugate plane of the spatiotemporal plane. Iris1 filters the spectrum to maintain a Gaussian mode (see more details in Supplementary Note 1). The *x*-polarized component of the object beam is modulated by a meticulously crafted hologram^67^ (see Methods section) to achieve the spatiotemporal LGst_0,1_ mode in 0th-order diffraction. The other diffraction orders are blocked by Iris2. In contrast, the residual *y*-polarized component is transmitted directly, maintaining a Gaussian shape. This arrangement ensures precise coaxial alignment between the *x*- and *y*-polarized components. The amplitude ratio and phase difference between them define the polarization state. In the LCP and RCP basis, a skyrmion topology is formed, while in the *x*- and *y*-polarization basis, a bimeron topology is realized. Here, the *x*- and *y*-polarizations are converted into RCP and LCP after passing through QWP1, forming a skyrmion topology. To fully characterize the spatiotemporal skyrmion wavepacket, we employ temporally sliced off-axis interference technique^58,68^.

Briefly, the chirped probe pulse passes through a pulse compressor and a time delay line, then is de-chirped into a Fourier-transform-limited pulse (~120 fs). The time-delayed probe beam interferes with the object beam, generating interference fringes that encode the spatial complex field of the wavepacket at a certain temporal slice. Scanning the probe pulse’s time delay enables the reconstruction of the object wavepacket’s spatiotemporal profile from the delay-dependent fringes^51^. HWP2 aligns the probe polarization with the object beam to enable interference. The spatiotemporal profiles of the RCP and LCP components are obtained through two separate 3D diagnostic measurement.

**Fig. 4:**
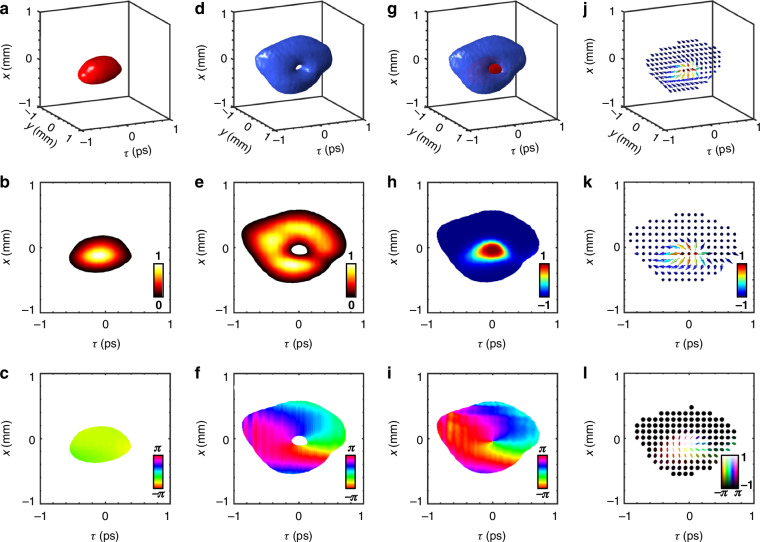
Experimentally synthesized spatiotemporal skyrmion wavepackets. **a**–**c** 3D iso-intensity (10% max) profiles and corresponding sliced intensity and phase patterns (at *y* = 0 plane) of the RCP component. **d**–**f** 3D iso-intensity (10% max) profiles and corresponding sliced intensity and phase patterns (at *y* = 0 plane) of the LCP component. **g** 3D iso-intensity (10% max) profiles of RCP and LCP components. The normalized Stokes distribution of **h** out-of-plane (S_3_) and **i** in-plane component [tan^−1^(S_1_/S_2_)]. **j** The 3D optical Stokes orientation distribution. **k** The top view of Stokes orientation distribution. **l** The elliptical polarization distributions of spatiotemporal skyrmion

We experimentally generated the fundamental Neel-type spatiotemporal Stokes skyrmion topology wavepacket, as shown in Fig. 4. The intensity iso-surface of the RCP component exhibits a Gaussian mode, as it is derived from the *y*-polarized component, which cannot be modulated by the SLM, as shown in Fig. 4a. The RCP component features a uniform sliced phase distribution [Fig. 4(b)] and a Gaussian-mode sliced amplitude profile [*y* = 0 plane, Fig. 4c], though with slight deformations. The deformations typically caused by the laser spectrum and optical system imperfections. Figure 4d presents the reconstructed spatiotemporal 3D iso-intensity surface of LCP component, indicating a complicated spatiotemporally coupled 3D wavepacket, which resembles a hollow doughnut-shaped structure. The sliced amplitude and phase distributions of the LCP component clearly exhibit its spatiotemporal vortex characteristics, including the hollow intensity profile and helical phase structure, as shown in Fig. 4e and 4f, respectively. A single-beam vector beam generation method, employing 0th-order diffraction complex amplitude holographic encoding, enables precise spatial coaxiality and temporal synchronization of the RCP and LCP components [Fig. 4g], yielding the designed polarization distribution depicted in Fig. 4l. The out-of-plane component *S*_3_ of the normalized Stokes parameters [Fig. 4h] exhibits a continuous variation from +1 at the core to –1 at the boundary, corresponding to the transition from the North Pole to the South Pole of the Poincaré sphere. Concurrently, the in-plane component tan^−1^(S_1_/S_2_)] [Fig. 4i] undergoes a phase evolution from 0 to 2*π*, encompassing the entire azimuthal range of the Poincaré sphere. The three-dimensional normalized Stokes field in spatiotemporal space spans the entire Poincaré sphere, constructing a skyrmion topology [Fig. 4j, k]. Despite slight distortions in the experimental results, the topological invariant remains preserved, with a skyrmion number of 0.92, highlighting the robustness of skyrmion topology. Additional spatiotemporal skyrmion topologies can be realized within this setup by adjusting the complex amplitude of the LCP component, as demonstrated through numerical simulations provided in Supplementary Note 2.

## Discussion

**Fig. 5:**
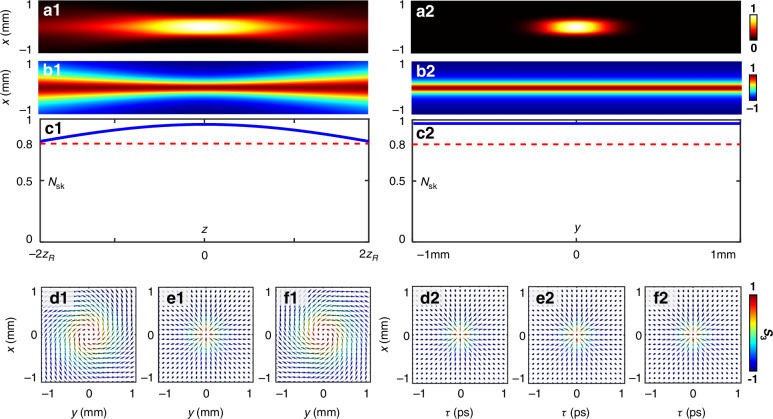
Comparison of skyrmion tubes in monochromatic and pulsed light. Skyrmion tubes of monochromatic light (**a1**–**f1**) and pulsed light (**a2**–**f2**). (**a1**, **a2**) Intensity distributions of the skyrmion tube for monochromatic light along the *z*-axis and pulsed light along the *y*-axis, respectively. **b1**, **b2** Out-of-plane Stokes orientation *s*_3_ distributions of the monochromatic and pulsed skyrmion tubes, respectively. **c1**, **c2** Skyrmion number of the monochromatic and pulsed skyrmion tubes along the *z*- and *y*-axis, respectively. **d1**–**f1** Helicity variations of the monochromatic skyrmion tube along the *z*-axis, caused by the Gouy phase shift during propagation. **d2**–**f2** Helicity of the pulsed skyrmion tube along the *y*-axis, which remains nearly unchanged

In monochromatic waves, the skyrmion plane (*x*, *y*) and the propagation dimension (*z*) define a time invariant, three-dimensional topological structure which can be trivially extended along the propagation direction, forming a skyrmion tube as shown in the left column of Fig. 5. While the topology remains invariant during propagation^29^, beam expansion pushes the intensity profile and *s*_3_ distribution toward the edges of the simulation domain as *z* increases. As a result, within a fixed observation window, the observed Skyrmion number exhibits a distance-dependent variation [Fig. 5a1–c1]. This variation is attributed to the longitudinal OAM along the *z*-dimension, which twists the iso-phase surface, inducing a Gouy phase shift that drives the helical twisting of the skyrmion tube [Fig. 5d1–f1]. In contrast, the intensity profile and *s*_3_ distributions (*τ-x* plane) for spatiotemporal skyrmion do not spread along *y* dimension due to the uniform vector sculpting applied consistently across *x-t* planes, as shown in Fig. 5a2, b2. The skyrmion number remains constant along the axis of skyrmion tube (Fig. 5(c2)). The transverse OAM also induces an invariant helicity [Fig. 5d2–f2], even as the spatial distribution of wavepacket change.

**Fig. 6:**
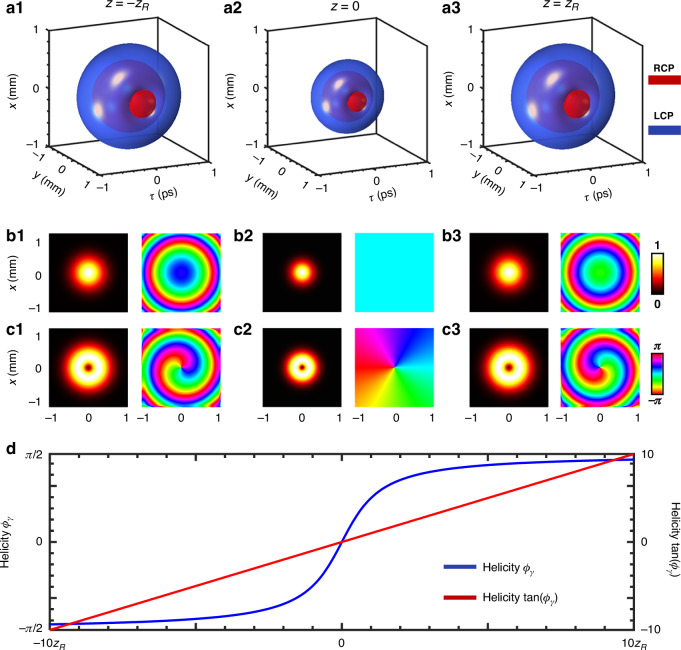
Numerical simulation of spatiotemporal skyrmion propagation in anomalous dispersive media. **a1**–**a3** The 3D iso-intensity (10% max) profiles of the RCP and LCP component of Spatiotemporal skyrmion at different propagation distances (–z_R_, 0, and z_R_). **b1**–**b3** and **c1**–**c3**) The corresponding amplitude and phase distributions of RCP and LCP component. **d** Evolution of skyrmion helicity along the propagation dimension z

The interplay between medium dispersion and spatial diffraction results in an intriguing propagation property for the spatiotemporal skyrmion. In dispersion-matched media, the propagation of spatiotemporal skyrmion pulses is governed by a precise balance between medium dispersion and spatial diffraction, leading to identical complex amplitude variations. Due to the form-invariance of LGst modes in dispersion-matched media, the beam spot size varies with propagation, as shown in Fig. 6a1–a3. The Gouy phase shift [Fig. 6b1–b3 and 6c1–c3] of skyrmion pulses causes a uniform variation in skyrmion helicity along the propagation distance *z* [Fig. 6d], while the *y*-dimension remains unaffected. At *z* = 0 plane, the skyrmion tube is of the Neel type, transitioning to an intermediate type as *z* approaches *z*_R_, and eventually becoming a Bloch type as *z* to infinity. In addition, the propagation of spatiotemporal skyrmion in free space could causes deformations in the skyrmion topology (as detailed in Supplementary Note 3).

The ability to scale the Skyrmion number is crucial for applications in topological information encoding and structured light-based data storage. In the spatiotemporal regime, the maximum achievable Skyrmion number is primarily limited by the highest-order spatiotemporal orbital angular momentum (ST-OAM) modes that can be stably generated and maintained. As the OAM order increases, the intensity ring radius also expands, making the measured Skyrmion number sensitive to the finite size of the observation region and more vulnerable to noise in low-intensity areas. To address these limitations, perfect vortex beams have been used to generate high-order Skyrmions with stable ring profiles^69^, while detection methods based on topological line integration of polarization fields offer improved robustness and accuracy compared to conventional gradient-based approaches^32^.

Spatiotemporal skyrmions offer several unique advantages and applications in the context of ultrafast optics and topological information processing^45,70^. By exploiting the temporal degree of freedom, they enable ultrafast polarization encoding and switching on picosecond or even sub-cycle timescales. This capability supports high-speed, topologically robust data transmission, making spatiotemporal skyrmions highly attractive for next-generation optical communication systems. Furthermore, incorporating time as an intrinsic coordinate grants access to higher-dimensional (3D space + 1D time) topologies, allowing the generation of complex structures such as arbitrary-order Hopfions^45,71,72^. This significantly broadens the scope of photonic topology and provides new insights into topological phenomena across diverse physical platforms. In addition, spatiotemporal skyrmions exhibit enhanced resilience to environmental perturbations; even when spatial modes are degraded, the temporal evolution of the Stokes vector can preserve the topological configuration, ensuring stable signal transmission under noisy or turbulent conditions.

In conclusion, we have extended skyrmion topology from the spatial to the spatiotemporal domain and demonstrated the generation of spatiotemporal skyrmions within ultrafast pulse wavepackets. These spatiotemporal skyrmion pulses, identified as vector solutions to Maxwell’s equations, exhibit a stable topological structure with polarization states evolving across both time and space. Experimentally, we construct spatiotemporal skyrmions by vectorially sculpting spatiotemporal wavepackets. Unlike conventional spatial skyrmions formed by longitudinal OAM, spatiotemporal skyrmions are induced by transverse OAM and exhibit no helical twisting perpendicular to the skyrmion plane, ensuring enhanced stability against deformation. Additionally, we show that the helicity of spatiotemporal skyrmions gradually evolves during propagation due to the interplay of medium dispersion and spatial diffraction. The ability to maintain a robust topological texture in the spatiotemporal domain opens new possibilities for structured light-matter interactions, optical information encoding, and ultrafast photonic applications. By advancing vectorial control to the spatiotemporal regime, this work broadens the scope of optical skyrmions and provides new opportunities for engineering topological states of light.

## Materials and methods

### The definition and calculation of the Stokes parameters

The Stokes parameters, as a pseudovector, characterize the polarization state of light under paraxial conditions (*E*_z_ = 0) and can be represented on the Poincaré sphere, where polarization states are mapped to specific points. For normalized Stokes parameters (*s*_1_, *s*_2_, *s*_3_), its direction generally aligns with the spatial coordinate axes (*x*, *y*, *z*). In the case of monochromatic light propagating along the *z*-axis, skyrmion topology can be constructed in the *x-y* plane. The *s*_3_ component corresponds to circular polarization states, with RCP aligned along +*z* and LCP along –*z*. Consequently, *s*_3_ is conventionally mapped to the *z*-axis, while *s*_1_ and *s*_2_ lie within the *x-y* plane.

To extend this concept to a pulsed wavepacket, the skyrmion topology is constructed in the *τ-x* plane using the normalized reduced Stokes parameters (*s*_1_, *s*_2_, *s*_3_) = (*S*_1_/S_0_, *S*_2_/S_0_, *S*_3_/S_0_). Here, *s*_3_ is mapped to the axis perpendicular to the topological plane (*τ*, *x*), while *s*_1_ and *s*_2_ is mapped along *τ*- and *x*-axis, respectively, providing an intuitive visualization of spatiotemporal skyrmion topology. The stokes parameter (*S*_0_, *S*_1_, *S*_2_, *S*_3_) can be calculated as follows:

4 $$\begin{array}{l}{S}_{0}={|{E}_{R}|}^{2}+{|{E}_{L}|}^{2}\\ {S}_{1}=2\mathrm{Re}({E}_{R}\cdot {E}_{L}^{\ast })\\ {S}_{2}=-2\text{Im}({E}_{R}\cdot {E}_{L}^{\ast })\\ {S}_{3}={|{E}_{R}|}^{2}-{|{E}_{L}|}^{2}\end{array}$$

Where the *E*_*R*_ and *E*_*L*_ represent the RCP and LCP components of the optical pulse wavepacket. The topological property of an optical skyrmion is determined by the skyrmion number, which represents the number of times the Stokes parameters (*s*_1_, *s*_2_, *s*_3_) wraps around a unit sphere, expressed as

5 $${N}_{s}=\frac{1}{4\pi }\iint {\bf{s}}\cdot \left(\frac{\partial {\bf{s}}}{\partial \xi_{0} }\times \frac{\partial {\bf{s}}}{\partial \eta_{0} }\right)d\xi_{0} d\eta_{0}$$

where *ξ*_0_ and *η*_0_ denote the orthogonal axis of the skyrmion plane.

### Parameters for spatiotemporal holographic pulse shaper

The laser source is a home-built Yb:fiber laser with an all-normal-dispersion (ANDi) configuration, delivering an output power of 60 mW. Its spectrum is centered at 1030 nm with a spectral bandwidth of 10 nm. The laser operates at a repetition rate of 20 MHz with a pulse duration of approximately 2 ps. At the output, the laser beam is expanded to a beam diameter of 2 mm to accommodate the long propagation distance in this experiment. With an overall transmission efficiency of about 1%, the resulting peak intensities remain well below nonlinear thresholds. Therefore, the spatiotemporal skyrmions are generated and observed entirely within the linear regime, with no detectable nonlinear distortion.

Based on the Fourier transform relationship between the space-time domain (*τ*, *x*) and the spatiotemporal frequency domain (*Ω*, *ξ*), we construct the spectrum in the spatiotemporal frequency domain to generate an LGst beam in the space-time domain (*τ*, *x*). The spatiotemporal Fourier transform is achieved by the spatially diffractive and temporally dispersive propagation, and then, a spatiotemporal LGst wavepacket is synthesized^53,59^.

In the vectorial spatiotemporal pulse shaper, the input 45-degree polarized beam is directed onto a reflective blazed grating (grating density of 1200 lines/mm, incident angle approximately 45°). The grating exhibits different diffraction efficiencies for *x*-polarized and x-polarized light, so the angle of HWP1 needs to be adjusted to modify the amplitude ratio between the *x*-polarized and *y*-polarized components. The first-order diffracted light is then collimated by a cylindrical lens (focal length 100 mm) onto the SLM screen (Model: Holoeye GAEA-2.1-NIR-069, 3840 × 2160 pixels, with a pixel pitch of 3.74 μm). The SLM modulates only the x-polarized component, which can be defined by the following expression

6 $$CGH=\,\mathrm{mod}\{\text{arg}(\psi )+{f}_{x}\xi \cdot {\rm{asinc}}(1-\left|\psi\right| )+\pi \cdot{\rm{asinc}}(\left|\psi\right| )+GDD\cdot {\Omega }^{2},2\pi \}$$

where, *f*_*x*_ represents the frequency of a linear phase ramp, with its depth determined by the modulus of the on-demand mode. Consequently, the undesired spatial-spectral energy is diffracted away from the optical axis by this phase grating after propagating a short distance from the SLM. A more detailed performance of the zero-order complex amplitude modulation can be found in Refs. ^52,53,67^.

## Supplementary information

Supplementary information of Construction of Optical Spatiotemporal Skyrmions

## Data Availability

The data that support the findings of this study are available from the corresponding author upon reasonable request.
