# Supplementary material for: Construction of optical spatiotemporal skyrmions
Source: Light Sci Appl. 2025 Sep 16;14:324. doi: 10.1038/s41377-025-02028-0 (PMC12441143; doi:10.1038/s41377-025-02028-0)
Supplement: Supplementary file 1 — Supplementary information of Construction of Optical Spatiotemporal Skyrmions [file 41377_2025_2028_MOESM1_ESM.docx]

Supplementary Information for

Construction of Optical Spatiotemporal Skyrmions

Houan Teng^1^, Xin Liu^1,2,3^, Nianjia Zhang^1^, Haihao Fan^1^, Guoliang Chen^1^, Qian Cao^1^, Jinzhan Zhong^1^, Xinrui Lei^1,*^, Qiwen Zhan^1,4,5,6,7,*^

^1^School of Optical-Electrical and Computer Engineering, University of Shanghai for Science and Technology, Shanghai 200093, China

^2^Shandong Provincial Engineering and Technical Center of Light Manipulations and Shandong Provincial Key Laboratory of Optics and Photonic Device, School of Physics and Electronics, Shandong Normal University, Jinan 250014, China.

^3^Collaborative Innovation Center of Light Manipulations and Applications, Shandong Normal University, Jinan 250358, China.

^4^Zhejiang Key Laboratory of 3D Micro/Nano Fabrication and Characterization, Department of Electronic and Information Engineering, School of Engineering, Westlake University, Hangzhou, Zhejiang 310030, China

^5^Westlake Institute for Optoelectronics, Fuyang, Hangzhou·311421, China.

^6^Zhangjiang Laboratory, 100 Haike Road, Shanghai 201204, China

^7^International Institute for Sustainability with Knotted Chiral Meta Matter (WPI-SKCM2), Hiroshima University, Higashihiroshima, Hiroshima, 739-8526, Japan.

*Corresponding authors: [xrlei@usst.edu.cn](mailto:xrlei@usst.edu.cn); [qwzhan@usst.edu.cn](mailto:qwzhan@usst.edu.cn)

Supplementary Note 1: Characterization of the light source's spectrum and pulse shape

The laser source is a home-built Yb:fiber laser with an all-normal-dispersion (ANDi) configuration. The laser spectrum is centered at 1030 nm with a spectral bandwidth of 10 nm, as shown in Fig. S1(a), and the corresponding 3D pulse envelope is depicted in Fig. S1(b). Due to significant chirp, the spectral shape and the sliced intensity distribution are very similar (bimodal mode), as shown in Fig. S1(a) and Fig. S1(c), respectively. The sliced phase pattern is presented in Fig. S1(d). To achieve a Gaussian profile for both the spectrum and pulse shape, Iris1 filters the spectrum to maintain a Gaussian mode (see main text). The bottom two rows illustrate the effects of spectral filtering: the second row shows the result of an incomplete filter, yielding a spectrum closer to Gaussian but still imperfect, while the third row demonstrates a complete filter, producing a nearly Gaussian spectrum and pulse shape that meet the requirements of this work.


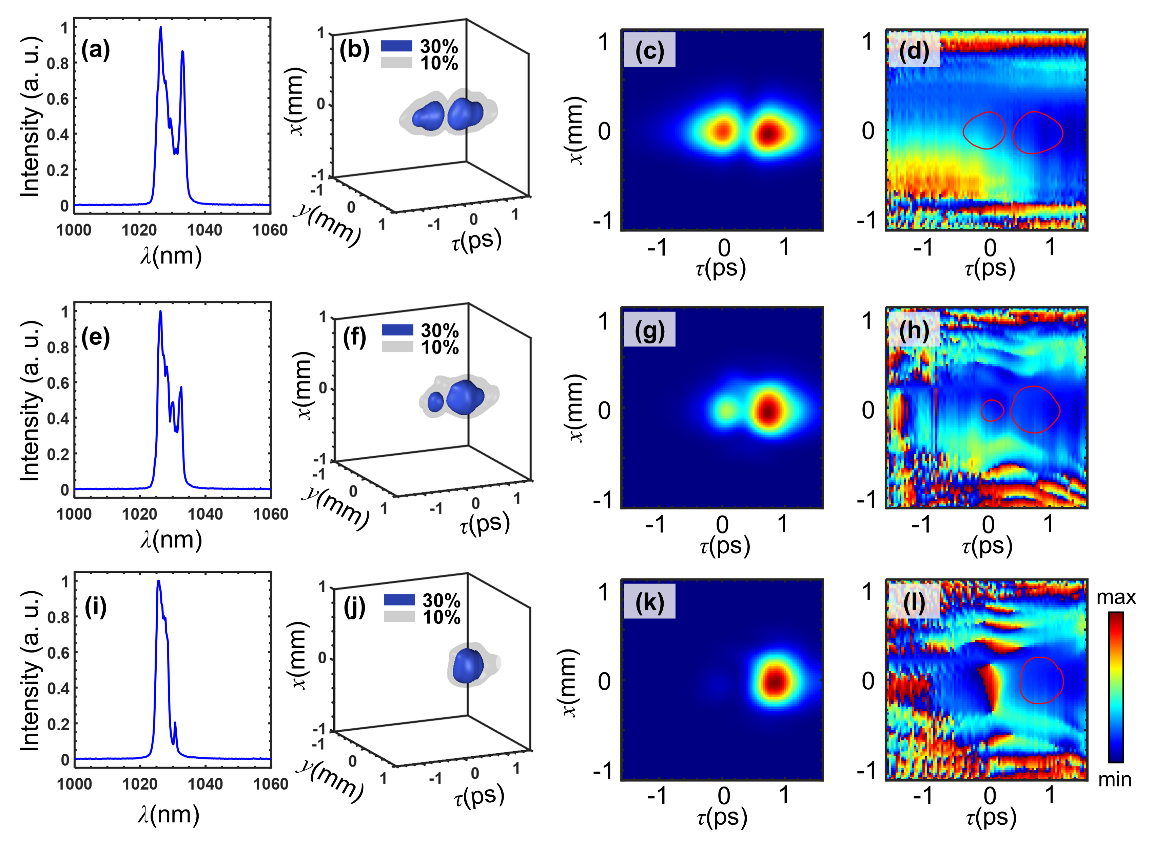


Fig. S1(a–d) shows the spectrum, 3D intensity iso-surface, sliced amplitude distribution, and sliced phase distribution of the pulse laser. Fig. S1(e–h) presents the results after an incomplete filter, including the spectrum, 3D intensity iso-surface, sliced amplitude, and sliced phase distribution. While the spectrum is closer to Gaussian, it remains imperfect. Fig. S1(i–l) depicts the results of a complete filter, producing a nearly Gaussian spectrum and pulse shape that meet the requirements of this work. The red circles in the phase pattern indicate intensity iso-contours.

Supplementary Note 2: Different type of spatiotemporal skyrmion

Our method enables the generation of various types of spatiotemporal skyrmions. The simplest spatiotemporal skyrmion is the Néel-type skyrmion [Fig. S2(c1)], formed by combining a spatiotemporal Gaussian pulse of right-handed circular polarization (RCP) [Fig. S2(a1, b1)] with a spatiotemporal *LG*_0,1_ mode of left-handed circular polarization (LCP) [Fig. S2(a2, b2)]. Different types of skyrmions can be generated by maintaining the RCP component as a Gaussian wavepacket and adjusting the complex amplitude of the LCP component. For instance, varying the phase difference between the RCP and LCP components [Fig. S2(a3, b3)] controls the helicity of skyrmion, with Fig. S2(c3) showcasing a Bloch-type spatiotemporal skyrmion. Additionally, adjusting the topological charge of the LCP component enables the creation of higher-order skyrmions. For example, employing an *LG*_0,2_ mode for the LCP component produces second-order spatiotemporal skyrmions, as illustrated in Fig. S2(c3).


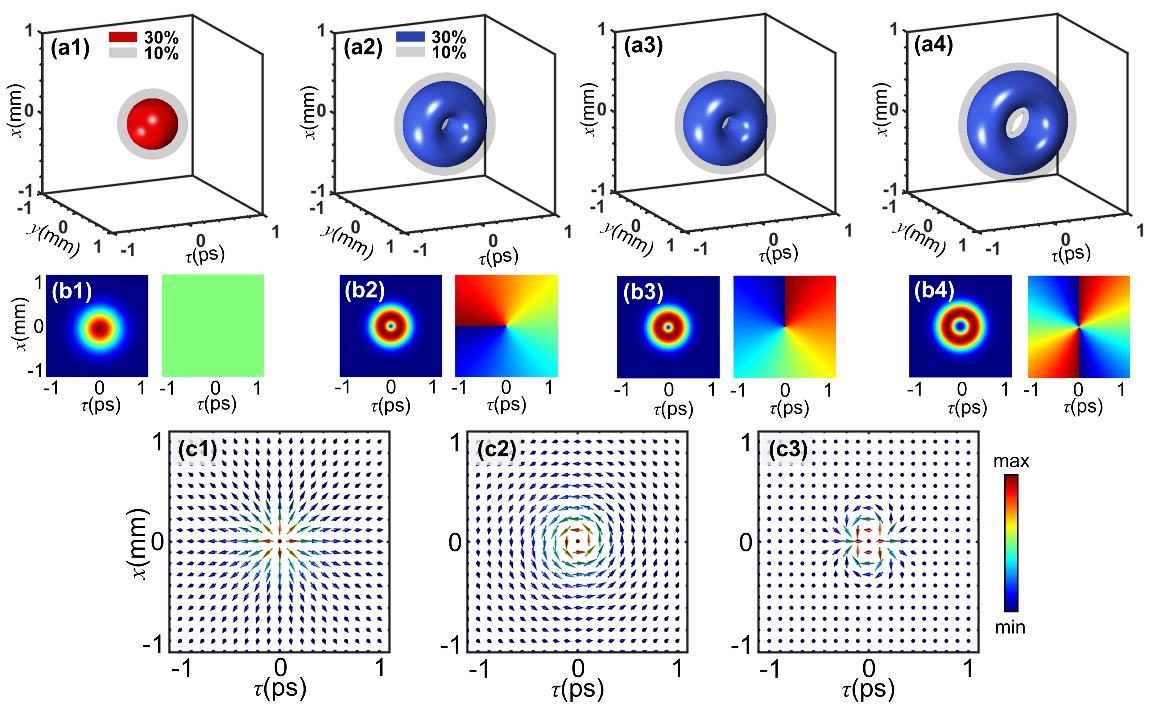


Fig. S2 Different types of spatiotemporal skyrmions. The first row shows the intensity iso-surface of the RCP component (a1) and LCP component (a2-a4) used to construct the spatiotemporal skyrmions. The second row presents the corresponding amplitude and phase distributions. The bottom row displays the Stokes vector orientation for the Néel-type spatiotemporal skyrmion (c1), Bloch-type spatiotemporal skyrmion (c2), and second-order spatiotemporal skyrmion (c3).

Supplementary Note 3: The propagation of spatiotemporal skyrmion in free space

The spatiotemporal evolution of a wavepacket in a dispersive medium can be numerically studied using the angular spectrum propagation theorem via the fast Fourier transform method, as described in [1–2].

$$\Psi\left( t,x,y,z \right)=\frac{e^{ik_{0}z}}{{8\pi}^{3}}\iiint\tilde{\Psi}\left( \Omega,k_{x},k_{y} \right)H\left( \Omega,k_{x},k_{y} \right)exp\left( -ik_{x}x-ik_{y}y-i\Omega t \right)d\Omega dk_{x}dk_{y}$$

(S1)

where *β*_2_$=-1/k_{0}$ is the group velocity dispersion coefficient of the dispersive medium. $H\left( \Omega,k_{x},k_{y} \right)=exp\left[ -i\left( k_{x}^{2}+k_{y}^{2} \right)z/2k_{0} \right]exp\left[ i\beta_{2}\Omega^{2}z/2 \right]$ is the transfer function of the dispersive medium. $\tilde{\Psi}\left( \Omega,k_{x},k_{y} \right)$ is the three-dimensional Fourier transform of $\Psi\left( t,x,y \right)$. *k_x_*, *k_y_* and $\Omega$are spatial frequencies in *x*, *y* directions and temporal frequencies in $t$ direction.

The skyrmions propagate in free space or in media with mismatched dispersion and diffraction could causes deformations in the skyrmion topology due to differing changes in dispersion along the *t*-dimension and diffraction along the *x-* and *y*-dimension, as shown in Fig. S3(a-c). Such deformations gradually affect the skyrmion number, altering the skyrmion topology Fig. S3(d1-d3). In the far field, the light field in the *t*-*x* plane splits into two halves, completely destroying the topological structure. Even when the observation window is enlarged to account for field expansion, the Skyrmion number still decreases during propagation, exhibiting a smooth transition from 1 to 0 as the beam evolves from the near-field to the far-field.

Such dispersion–diffraction mismatch in spatiotemporal skyrmions is analogous to the behavior of spatial skyrmions passing through astigmatic optical elements like cylindrical lenses. These lenses induce asymmetric focusing along one transverse axis, breaking the spatial symmetry and causing vortex splitting that degrades the skyrmion texture. Therefore, ensuring matched dispersion and diffraction conditions is crucial to maintain the topological integrity of both spatiotemporal and spatial skyrmions.


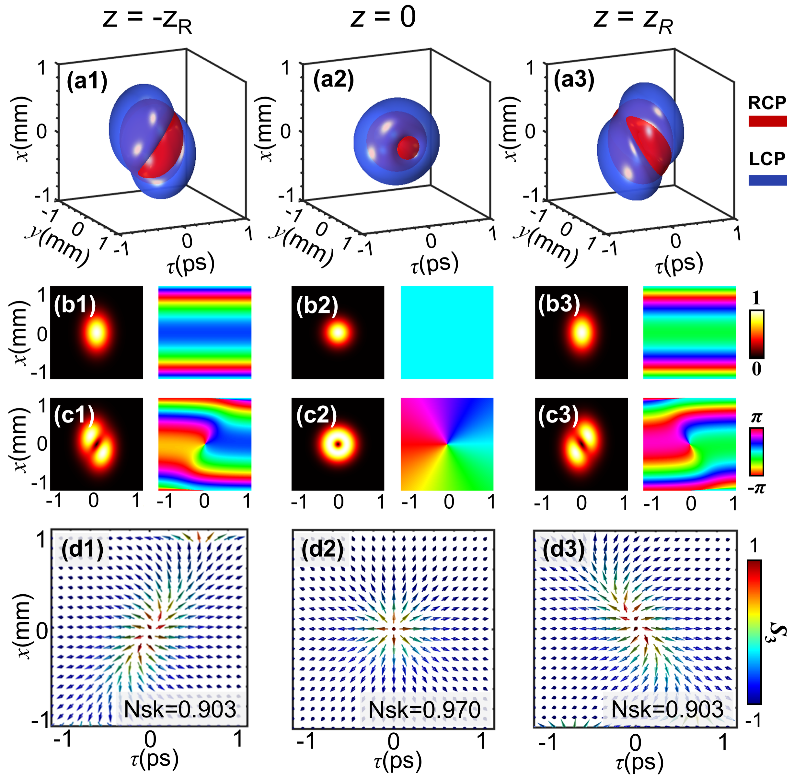


Fig. S3 Numerical simulation of spatiotemporal skyrmion propagation in free space. (a1-a3) The intensity iso-surfaces (10% max) at different propagation distances, (b, c) The corresponding amplitude and phase distributions. (d) The corresponding Stokes spatiotemporal skyrmion topology.

Supplementary References

1. Agrawal, G. P. Nonlinear Fiber Optics (Academic Press, 2012).
2. Schmidt, J. D. Numerical Simulation of Optical Wave Propagation with Examples in MATLAB (SPIE, 2010)
